# Supplementary material for: Genome Insights into Autopolyploid Evolution: A Case Study in Senecio doronicum (Asteraceae) from the Southern Alps
Source: Plants (Basel). 2022 May 2;11(9):1235. doi: 10.3390/plants11091235 (PMC9099586; doi:10.3390/plants11091235)
Supplement: Supplementary file 1 [file plants-11-01235-s001.zip › Supplementary Figures S1 and S2.pdf]

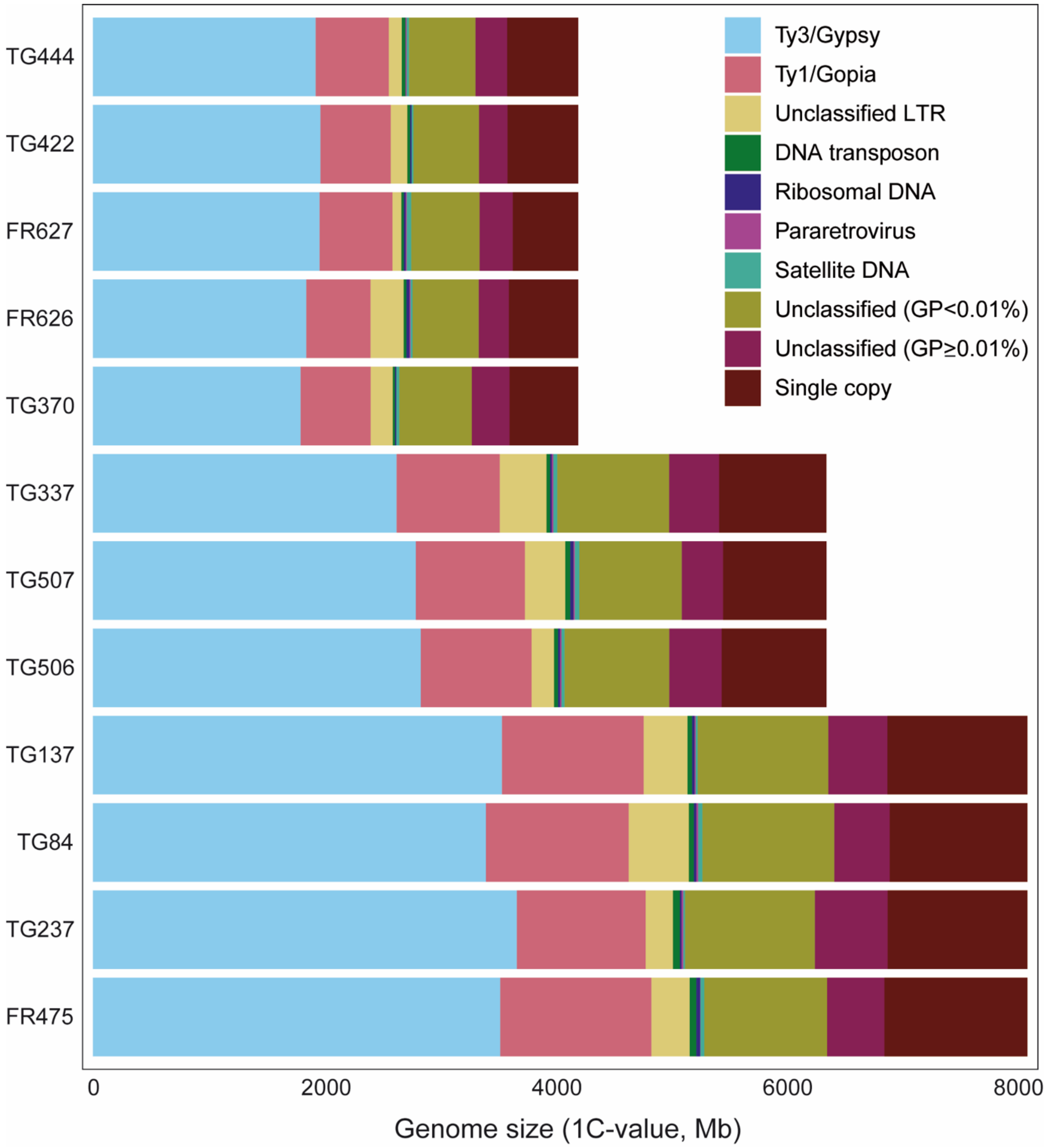

Supplementary Figure S1. Genomic composition of all *Senecio doronicum* accessions across cytotypes (4x, 6x and 8x). Estimates of the genomic abundances (in Mb/1C) of different repeats are indicated and colored by repeat class [GP = Genome proportion (%), LTR = Long Terminal Repeat].

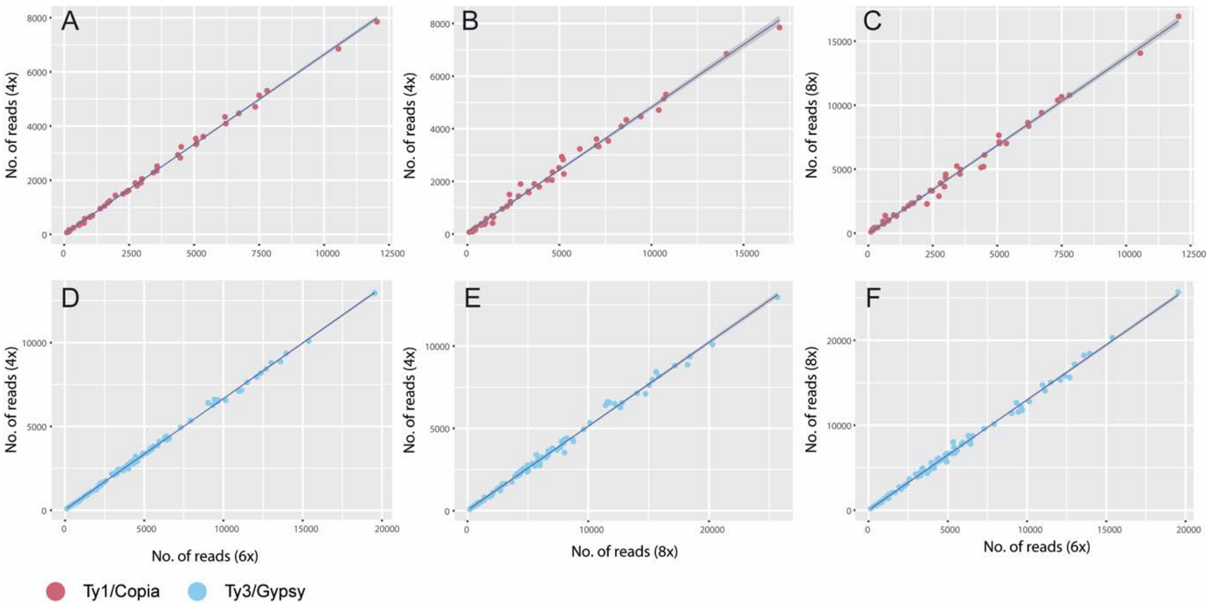

Supplementary Figure S2. Pairwise scatterplot comparisons of the number of reads included in repeat clusters from each cytotype. The slope indicates the genome size ratio between each cytotype. (**A**–**C**) Ty1/Copia. (**D**–**F**) Ty3/Gypsy.
