# Supplementary material for: Genome Insights into Autopolyploid Evolution: A Case Study in Senecio doronicum (Asteraceae) from the Southern Alps
Source: Plants (Basel). 2022 May 2;11(9):1235. doi: 10.3390/plants11091235 (PMC9099586; doi:10.3390/plants11091235)
Supplement: Supplementary file 1 [file plants-11-01235-s001.zip › Supplementary Table S1.pdf]

Supplementary Table S1. Cytotype screening of *Senecio doronicum* individuals from Tête Grosse (Alps du Aute Provence. Internal standard used in flow cytometry: *Petroselinum crispum* 'Champion Curler Moss' (2C = 4.45 pg).

Sample ID (Code TG)	FCM ratio (standard vs. Sample)	Genome size (2C, pg)	DNA Ploidy
1R	3.748	16.679	8x
2R	3.718	16.545	8x
3R	3.665	16.309	8x
4R	3.783	16.834	8x
5R	3.679	16.372	8x
6R	3.688	16.412	8x
7R	3.723	16.567	8x
8R	3.721	16.558	8x
9	3.763	16.745	8x
10	3.763	16.745	8x
11	3.763	16.745	8x
12	3.763	16.745	8x
13	3.763	16.745	8x
14R	3.715	16.532	8x
15	3.788	16.857	8x
16	3.788	16.857	8x
18R	3.769	16.772	8x
19	3.788	16.857	8x
20	3.788	16.857	8x
21	3.788	16.857	8x
22	3.730	16.599	8x
23	3.730	16.599	8x
24R	3.801	16.914	8x
25	3.730	16.599	8x
26	3.730	16.599	8x
27	3.730	16.599	8x
28R	3.775	16.799	8x
29	3.810	16.955	8x
30	3.810	16.955	8x
31	3.810	16.955	8x
32	3.810	16.955	8x
33	3.810	16.955	8x
34	3.867	17.208	8x
35	3.867	17.208	8x
36	3.867	17.208	8x
37	3.867	17.208	8x
38	3.867	17.208	8x

39R	3.720	16.554	8x
41R	3.683	16.389	8x
43	3.804	16.928	8x
44	3.804	16.928	8x
46	3.804	16.928	8x
47R	3.785	16.843	8x
48	3.835	17.066	8x
51	3.590	15.976	8x
52	3.572	15.895	8x
53	3.572	15.895	8x
54	3.497	15.562	8x
55	3.497	15.562	8x
56	3.497	15.562	8x
57	3.497	15.562	8x
58	3.497	15.562	8x
59	3.502	15.584	8x
60	3.502	15.584	8x
61R	3.735	16.621	8x
62	3.502	15.584	8x
63	3.502	15.584	8x
64R	3.745	16.665	8x
65	3.502	15.584	8x
66	3.593	15.989	8x
67	3.593	15.989	8x
68	3.593	15.989	8x
69	3.593	15.989	8x
70R	3.550	15.798	8x
71	3.593	15.989	8x
72	3.540	15.753	8x
73	3.540	15.753	8x
74	3.540	15.753	8x
75	3.540	15.753	8x
76	3.540	15.753	8x
77	3.609	16.060	8x
78	3.609	16.060	8x
79	3.609	16.060	8x
80	3.609	16.060	8x
81	3.609	16.060	8x
82	3.473	15.455	8x
83	3.473	15.455	8x
84	3.473	15.455	8x
85	3.473	15.455	8x
86	3.473	15.455	8x
87	3.572	15.895	8x
88	3.572	15.895	8x
89	3.590	15.976	8x

90	3.590	15.976	8x
91	3.590	15.976	8x
92	3.590	15.976	8x
93	3.595	15.998	8x
94	3.572	15.895	8x
95	3.497	15.562	8x
96	3.497	15.562	8x
97	3.497	15.562	8x
98	3.497	15.562	8x
99	3.497	15.562	8x
100R	3.721	16.558	8x
101R	3.827	17.030	8x
103	3.578	15.922	8x
104	3.578	15.922	8x
105	3.578	15.922	8x
106	3.578	15.922	8x
107	3.578	15.922	8x
108	3.639	16.194	8x
109	3.639	16.194	8x
110	3.639	16.194	8x
111	3.639	16.194	8x
112	3.639	16.194	8x
113	3.603	16.033	8x
114	3.603	16.033	8x
115	3.603	16.033	8x
115	1.998	8.891	4x
116	3.603	16.033	8x
117	3.603	16.033	8x
118	3.563	15.855	8x
119	3.563	15.855	8x
120RA	3.775	16.799	8x
120RB	3.817	16.986	8x
121R	3.804	16.928	8x
122	3.563	15.855	8x
123	3.563	15.855	8x
124	3.719	16.550	8x
125	3.719	16.550	8x
126	3.719	16.550	8x
127	3.719	16.550	8x
128	3.719	16.550	8x
129	3.766	16.759	8x
130	3.766	16.759	8x
131	3.766	16.759	8x
132	3.766	16.759	8x
133	3.766	16.759	8x
134	3.700	16.465	8x

135	3.700	16.465	8x
136	3.700	16.465	8x
137	3.700	16.465	8x
138R	3.725	16.576	8x
139R	3.867	17.208	8x
141	3.734	16.616	8x
142	3.734	16.616	8x
143	3.734	16.616	8x
144	3.734	16.616	8x
145R	3.854	17.150	8x
146	3.852	17.141	8x
147	3.852	17.141	8x
148	3.852	17.141	8x
149	3.852	17.141	8x
150	3.852	17.141	8x
151	3.690	16.421	8x
152	3.690	16.421	8x
153	3.690	16.421	8x
154	3.690	16.421	8x
155	3.721	16.558	8x
156	3.721	16.558	8x
157	3.721	16.558	8x
158	3.721	16.558	8x
159	3.721	16.558	8x
161	3.660	16.287	8x
162	3.660	16.287	8x
163	3.660	16.287	8x
164R	3.779	16.817	8x
165R	3.780	16.821	8x
166	3.660	16.287	8x
167	3.743	16.656	8x
168	3.743	16.656	8x
169	3.743	16.656	8x
170	3.743	16.656	8x
171	3.743	16.656	8x
172R	3.706	16.492	8x
173R	3.806	16.937	8x
174R	3.699	16.461	8x
175	3.677	16.363	8x
176	3.677	16.363	8x
177	3.677	16.363	8x
178	3.677	16.363	8x
179	3.677	16.363	8x
180	3.633	16.167	8x
181	3.633	16.167	8x
182	3.633	16.167	8x

183	3.633	16.167	8x
184R	3.782	16.830	8x
186	3.633	16.167	8x
187	3.737	16.630	8x
188	3.737	16.630	8x
189	3.737	16.630	8x
190	3.737	16.630	8x
191	3.737	16.630	8x
192	3.460	15.397	8x
193	3.460	15.397	8x
194	3.460	15.397	8x
195	3.460	15.397	8x
196	3.460	15.397	8x
197	3.571	15.891	8x
198	3.571	15.891	8x
199	3.571	15.891	8x
200	3.494	15.548	8x
202	3.478	15.477	8x
203	3.472	15.450	8x
204	3.472	15.450	8x
205	3.500	15.575	8x
206	3.494	15.548	8x
207	3.612	16.073	8x
208	3.478	15.477	8x
209	3.574	15.904	8x
210	3.574	15.904	8x
211	3.612	16.073	8x
212	3.567	15.873	8x
213	3.654	16.260	8x
214	3.654	16.260	8x
215	3.500	15.575	8x
216	3.654	16.260	8x
217	3.571	15.891	8x
218	3.743	16.656	8x
219	3.571	15.891	8x
220	3.574	15.904	8x
221	3.494	15.548	8x
222	3.724	16.572	8x
223	3.457	15.384	8x
224	3.472	15.450	8x
225	3.574	15.904	8x
226	3.457	15.384	8x
227	3.472	15.450	8x
228	3.654	16.260	8x
229	3.567	15.873	8x
230	3.567	15.873	8x

231	3.743	16.656	8x
232	3.478	15.477	8x
233	3.567	15.873	8x
234	3.500	15.575	8x
235	3.478	15.477	8x
236	3.457	15.384	8x
237	3.457	15.384	8x
238	3.654	16.260	8x
239	3.457	15.384	8x
240	3.494	15.548	8x
241	3.472	15.450	8x
242	3.494	15.548	8x
243	3.478	15.477	8x
244	3.500	15.575	8x
245	3.500	15.575	8x
246	3.612	16.073	8x
248	3.612	16.073	8x
249	3.612	16.073	8x
250	3.724	16.572	8x
251	3.793	16.879	8x
252	3.724	16.572	8x
253	3.766	16.759	8x
254	3.793	16.879	8x
256	3.724	16.572	8x
257	3.766	16.759	8x
258	3.766	16.759	8x
259	3.793	16.879	8x
260	3.743	16.656	8x
261	3.754	16.705	8x
270	3.754	16.705	8x
271	3.766	16.759	8x
272	3.766	16.759	8x
274	3.743	16.656	8x
275	3.724	16.572	8x
277	3.754	16.705	8x
278	3.754	16.705	8x
279	3.793	16.879	8x
280	3.754	16.705	8x
301R	3.903	17.368	8x
302R	3.938	17.524	8x
303R	3.705	16.487	8x
304R	3.882	17.275	8x
306R	3.874	17.239	8x
307R	3.789	16.861	8x
308	3.864	17.195	8x
309	3.759	16.728	8x

310R	3.658	16.278	8x
311	3.752	16.696	8x
312	3.984	17.729	8x
313	3.705	16.487	8x
315	3.690	16.421	8x
318	3.508	15.611	8x
320	3.787	16.852	8x
322	3.865	17.199	8x
324	3.853	17.146	8x
325	3.776	16.803	8x
326	3.774	16.794	8x
327R	3.732	16.607	8x
328	3.744	16.661	8x
329R	2.039	9.074	4x
333	3.609	16.060	8x
335R	3.980	17.711	8x
337R	2.888	12.852	6x
338R	3.686	16.403	8x
340R	3.792	16.874	8x
342	3.706	16.492	8x
343R	2.023	9.002	4x
344R	1.976	8.793	4x
345R	2.046	9.105	4x
347	2.012	8.953	4x
348R	2.058	9.158	4x
351R	3.816	16.981	8x
352	3.668	16.323	8x
353	3.789	16.861	8x
354	3.516	15.646	8x
355R	2.008	8.936	4x
357R	2.014	8.962	4x
358	2.034	9.051	4x
359	2.003	8.913	4x
360	1.930	8.589	4x
362	2.057	9.154	4x
363R	2.068	9.203	4x
364	2.024	9.007	4x
365R	2.024	9.007	4x
366R	2.036	9.060	4x
367R	2.009	8.940	4x
368	1.997	8.887	4x
369	1.973	8.780	4x
370	2.018	8.980	4x
371	1.988	8.847	4x
372	2.043	9.091	4x
373	2.041	9.082	4x

374	2.009	8.940	4x
377	3.878	17.257	8x
380	3.872	17.230	8x
381	3.756	16.714	8x
387	3.900	17.355	8x
388	3.742	16.652	8x
389	3.745	16.665	8x
390	3.688	16.412	8x
391	3.724	16.572	8x
392	3.627	16.140	8x
392	3.832	17.052	8x
393	3.690	16.421	8x
394	3.716	16.536	8x
395	3.820	16.999	8x
396	3.753	16.701	8x
397	3.832	17.052	8x
398	3.767	16.763	8x
399	3.519	15.660	8x
400	3.747	16.674	8x
401	3.529	15.704	8x
403	1.979	8.807	4x
406	3.737	16.630	8x
408	3.468	15.433	8x
409	3.704	16.483	8x
410	3.823	17.012	8x
411	3.570	15.887	8x
412	1.998	8.891	4x
413R	2.006	8.927	4x
414	3.711	16.514	8x
415	2.062	9.176	4x
416	2.048	9.114	4x
417	2.044	9.096	4x
420	2.016	8.971	4x
421	1.905	8.477	4x
422	2.007	8.931	4x
423	1.987	8.842	4x
424	1.969	8.762	4x
426	2.007	8.931	4x
427	2.028	9.025	4x
428	2.016	8.971	4x
430	1.958	8.713	4x
431	1.872	8.330	4x
432R	2.035	9.056	4x
439R	1.952	8.686	4x
442	2.015	8.967	4x
443R	1.959	8.718	4x

444	2.000	8.900	4x
445	2.008	8.936	4x
446	2.028	9.025	4x
447	2.000	8.900	4x
448	1.963	8.735	4x
449	2.038	9.069	4x
450	1.968	8.758	4x
453	2.046	9.105	4x
456	2.003	8.913	4x
457	2.032	9.042	4x
458	2.013	8.958	4x
459	2.054	9.140	4x
460R	2.059	9.163	4x
462	2.014	8.962	4x
463	1.963	8.735	4x
464	1.950	8.678	4x
465	2.015	8.967	4x
466	2.022	8.998	4x
467	2.009	8.940	4x
468R	2.041	9.082	4x
470	2.038	9.069	4x
471	2.005	8.922	4x
472	2.001	8.904	4x
473	2.029	9.029	4x
474R	1.994	8.873	4x
475	1.977	8.798	4x
476	2.011	8.949	4x
477	1.997	8.887	4x
478	2.024	9.007	4x
479	2.036	9.060	4x
480	1.976	8.793	4x
481	2.000	8.900	4x
482	2.001	8.904	4x
484	2.050	9.123	4x
485	2.018	8.980	4x
486	2.020	8.989	4x
489	2.023	9.002	4x
490	2.009	8.940	4x
491	1.996	8.882	4x
493	2.035	9.056	4x
494	2.017	8.976	4x
495	1.973	8.780	4x
496	1.996	8.882	4x
497	1.986	8.838	4x
498	2.032	9.042	4x
499	1.935	8.611	4x

500	2.054	9.140	4x
501	2.038	9.069	4x
502	1.952	8.686	4x
503	1.945	8.655	4x
504	1.903	8.468	4x
505	1.983	8.824	4x
506	2.794	12.433	6x
507	2.755	12.260	6x
508	2.041	9.082	4x
509	1.946	8.660	4x
510	1.946	8.660	4x
511	1.946	8.660	4x
513	1.946	8.660	4x
514	1.907	8.486	4x
515	2.017	8.976	4x
516	2.040	9.078	4x
517	1.963	8.735	4x
518	1.998	8.891	4x
519	2.027	9.020	4x
520	1.975	8.789	4x
521R	1.970	8.767	4x
522	2.035	9.056	4x
523	1.963	8.735	4x
524	1.981	8.815	4x
526	2.058	9.158	4x
527	2.058	9.158	4x
528	3.765	16.754	8x
529R	2.056	9.149	4x
530	2.076	9.238	4x
531	1.992	8.864	4x
532	2.028	9.025	4x
535	3.685	16.398	8x
539R	2.000	8.900	4x
540	3.777	16.808	8x
541	3.708	16.501	8x
542	3.708	16.501	8x
543	3.765	16.754	8x
544	3.744	16.661	8x
545	2.012	8.953	4x
546	3.584	15.949	8x
548	3.763	16.745	8x
549R	2.013	8.958	4x
550	3.687	16.407	8x
551	2.027	9.020	4x
552	2.034	9.051	4x
553	3.620	16.109	8x

554	3.643	16.211	8x
555	3.887	17.297	8x
556	3.758	16.723	8x
558	3.830	17.044	8x
559	3.733	16.612	8x
560a	1.976	8.793	4x
560b	3.841	17.092	8x
561	1.978	8.802	4x
563	3.857	17.164	8x
565	3.533	15.722	8x
567R	1.983	8.824	4x
568	3.701	16.469	8x
570	3.971	17.671	8x
571	3.757	16.719	8x
572	1.952	8.686	4x
573	3.742	16.652	8x
574	3.753	16.701	8x
576	3.709	16.505	8x
578	3.808	16.946	8x
579	3.742	16.652	8x
580	3.800	16.910	8x
583	3.759	16.728	8x
585	3.944	17.551	8x
586	3.902	17.364	8x
591	3.727	16.585	8x
592	3.716	16.536	8x
593	4.011	17.849	8x
594	3.793	16.879	8x
595	3.848	17.124	8x
597	3.551	15.802	8x
598	3.633	16.167	8x
599	3.764	16.750	8x
600	3.662	16.296	8x
601	3.730	16.599	8x
602	2.002	8.909	4x
603	3.716	16.536	8x
604	1.967	8.753	4x
605	2.029	9.029	4x
606	2.039	9.074	4x
607	2.032	9.042	4x
608	2.056	9.149	4x
609	1.975	8.789	4x
610	2.003	8.913	4x
613	3.691	16.425	8x
700R	2.040	9.078	4x
702	1.971	8.771	4x

703	1.946	8.660	4x
703	1.973	8.780	4x
705	2.058	9.158	4x
