# Supplementary material for: Genome Insights into Autopolyploid Evolution: A Case Study in Senecio doronicum (Asteraceae) from the Southern Alps
Source: Plants (Basel). 2022 May 2;11(9):1235. doi: 10.3390/plants11091235 (PMC9099586; doi:10.3390/plants11091235)
Supplement: Supplementary file 1 [file plants-11-01235-s001.zip › Supplementary Table S2.pdf]

Supplementary Table S2. Cytotype screening of *Senecio doronicum* individuals from outside Tête Grosse (Alps du Aute Provence). Internal standard used in flow cytometry: *Petroselinum crispum* 'Champion Curler Moss' (2C = 4.45 pg).

Sample ID	FCM ratio (standard vs. Sample)	Genome size (2C, pg)	DNA Ploidy	Lat. N	Long. E
FR356	3.656	16.27	8x	44.33665	6.28209
FR357	3.697	16.45	8x	44.33665	6.28209
FR358	3.753	16.7	8x	44.33462	6.29482
FR359	3.802	16.92	8x	44.33462	6.29482
FR360	3.771	16.78	8x	44.3405	6.29449
FR361	3.854	17.15	8x	44.3405	6.29449
FR363	3.737	16.63	8x	44.40339	6.37521
FR364	3.654	16.26	8x	44.39758	6.38375
FR365	3.584	15.95	8x	44.39758	6.38375
FR379	3.717	16.54	8x	44.40943	6.38709
FR380	3.715	16.53	8x	44.40943	6.38709
FR386	3.742	16.65	8x	44.332	6.29235
FR387	3.665	16.31	8x	44.332	6.29235
FR417	3.654	16.26	8x	44.31216	6.43589
FR435	3.910	17.4	8x	44.28397	6.43458
FR459	3.807	16.94	8x	44.72351	6.31857
FR463	3.757	16.72	8x	44.72623	6.32373
FR472	3.818	16.99	8x	44.72993	6.32625
FR474	3.838	17.08	8x	44.72324	6.3443
FR475	3.822	17.01	8x	44.72992	6.32625
FR476	3.791	16.87	8x	44.72099	6.34373
FR478	3.784	16.84	8x	44.72046	6.34202
FR479	3.766	16.76	8x	44.72046	6.34202
FR482	3.816	16.98	8x	44.57619	6.33727
FR485	3.865	17.2	8x	44.58065	6.33232
FR601	3.719	16.55	8x	44.06522	6.40574
FR626	1.921	8.55	4x	44.25249	6.71275
FR627	1.935	8.61	4x	44.25315	6.71517
FR653	1.966	8.75	4x	44.3197	6.69477
FR654	1.989	8.85	4x	44.31719	6.690034
FR671	3.625	16.13	8x	44.20338	7.1513
FR696	3.510	15.62	8x	44.34501	6.80097
FR711a	3.589	15.97	8x	44.32002	6.43188
FR715	3.782	16.83	8x	44.33681	6.2822
FR720	3.546	15.78	8x	44.3342	6.29422
FR721	3.578	15.92	8x	44.34052	6.29454
