# Supplementary material for: Genome Insights into Autopolyploid Evolution: A Case Study in Senecio doronicum (Asteraceae) from the Southern Alps
Source: Plants (Basel). 2022 May 2;11(9):1235. doi: 10.3390/plants11091235 (PMC9099586; doi:10.3390/plants11091235)
Supplement: Supplementary file 1 [file plants-11-01235-s001.zip › Supplementary Table S3.pdf]

Supplementary Table S3. Details per individual of genome skimming and individual clustering analysis of specimens analysed from Tête Grosse (TG) and outside this population (FR).

Individual code	GS 1C (Mb)	Chrom. number (2n)	Sequencing run number	Individual clustering	
				No. reads	Coverage (× 1C)
TG84	8,097.84	80	SAMN25529504	1,200,000	0.022 ×
TG137	8,097.84	80	SAMN25529505	1,200,000	0.022 ×
TG237	8,097.84	80	SAMN25529506	1,200,000	0.022 ×
FR475	8,097.84	80	SAMN25529513	1,200,000	0.022 ×
TG337R	6,357	60	SAMN25529507	1,200,000	0.028 ×
TG506	6,357	60	SAMN25529511	1,200,000	0.028 ×
TG507	6,357	60	SAMN25529512	1,200,000	0.028 ×
TG370	4,205.4	40	SAMN25529508	1,200,000	0.043 ×
TG422	4,205.4	40	SAMN25529509	1,200,000	0.043 ×
TG444	4,205.4	40	SAMN25529510	1,200,000	0.043 ×
FR626	4,205.4	40	SAMN25529514	1,200,000	0.043 ×
FR627	4,205.4	40	SAMN25529515	1,200,000	0.043 ×
