# Supplementary material for: Genome Insights into Autopolyploid Evolution: A Case Study in Senecio doronicum (Asteraceae) from the Southern Alps
Source: Plants (Basel). 2022 May 2;11(9):1235. doi: 10.3390/plants11091235 (PMC9099586; doi:10.3390/plants11091235)
Supplement: Supplementary file 1 [file plants-11-01235-s001.zip › Supplementary Table S5.pdf]

Supplementary Table S5. Comparison of genome compositions between individuals of the same cytotype using paired Wilcoxon tests. All samples were from Tete Grosse with the exception of individuals FR626/FR627 and FR475, which were from Allos and Orciéres respectively.

Cytotype	Comparison	Wilcoxon test statistic (V)	p-value
**Tetraploids**	TG370-TG422	43	0.791
	TG370-TG444	44	0.733
	TG370-FR626	31	0.569
	TG370-FR627	53	0.3
	TG444-FR626	35	0.791
	TG444-TG422	43	0.791
	TG444-FR627	27	0.38
	TG422-FR626	26	0.339
	TG422-FR627	42	0.85
	FR627-FR626	32	0.622
**Hexaploids**	TG337R-TG506	44	0.733
	TG337R-TG507	47	0.569
	TG506-TG507	43	0.791
**Octoploids**	TG84-TG137	39	0.625
	TG84-TG237	44	0.35
	TG84-FR475	39	0.625
	TG237-FR475	35	0.894
	TG237-TG137	24	0.45
	TG137-FR475	38	0.689
